# Gene Expression, Bacteria Viability and Survivability Following Spray Drying of *Mycobacterium smegmatis*

**DOI:** 10.3390/ma3042684

**Published:** 2010-04-13

**Authors:** Elizabeth Hunter Lauten, Brian L. Pulliam, Jessica DeRousse, Deen Bhatta, David A. Edwards

**Affiliations:** 1 Harvard School of Engineering and Applied Sciences, 58 Oxford Street, ESL 406, Cambridge, MA 02138, USA; E-Mails: lauteneh@seas.harvard.edu (E.H.L.); jderouss@seas.harvard.edu (J.D.); dbhatta@seas.harvard.edu (D.B.); 2 Harvard School of Engineering and Applied Sciences, 29 Oxford Street, 322 Pierce Hall, Cambridge, MA 02138, USA; 3 Wyss Institute of Biologically Inspired Engineering, Harvard University HIM, 10th Floor,4 Blackfan Circle, Boston, MA 02115, USA

**Keywords:** mycobacterium, gene expression, thermostability

## Abstract

We find that *Mycobacterium* *smegmatis* survives spray drying and retains cell viability in accelerated temperature stress (40 °C) conditions with a success rate that increases with increasing thermal, osmotic, and nutrient-restriction stresses applied to the mycobacterium prior to spray drying. *M.* *smegmatis* that are spray dried during log growth phase, where they suffer little or no nutrient-reduction stress, survive for less than 7 days in the dry powder state at accelerated temperature stress conditions, whereas *M.* *smegmatis* that are spray dried during stationary phase, where cells do suffer nutrient reduction, survive for up to 14 days. *M. smegmatis* that are spray dried from stationary phase, subjected to accelerated temperature stress conditions, regrown to stationary phase, spray dried again, and resubmitted to this same process four consecutive times, display, on the fourth spray drying iteration, an approximate ten-fold increase in stability during accelerated temperature stress testing, surviving up to 105 days. Microarray tests revealed significant differences in genetic expression of *M.* *smegmatis* between log phase and stationary phase conditions, between naïve (non spray-dried) and multiply cycled dried *M.* *smegmatis* (in log and stationary phase), and between *M. smegmatis* in the dry powder state following a single spray drying operation and after four consecutive spray drying operations. These differences, and other phenotypical differences, point to the carotenoid biosynthetic pathway as a probable pathway contributing to bacteria survival in the spray-dried state and suggests strategies for spray drying that may lead to significantly greater room-temperature stability of mycobacteria, including mycobacterium *bovis* bacille Calmette-Guerin (BCG), the current TB vaccine.

## 1. Introduction

Tuberculosis kills more than three million people annually and is ranked among the top ten causes of global mortality and morbidity [1]. The current Mycobacterium *bovis* bacille Calmette-Guerin (BCG) TB vaccine, which is given intradermally to 100 million infants annually, is formulated as a dry powder via freeze drying (lyophilization) [2,3]. This process typically results in a live attenuated vaccine with 10–30% viability relative to the pre-dried formulation [4]. When kept at refrigerated conditions the commercial lyophilized BCG loses approximately one log of activity after one year to 18 months on the shelf. This is dramatically reduced when placed at room temperature stability conditions (25 °C) resulting in a month or two of accepted viability [1]. Preserving the viability of BCG in dried powders is thought to be an important factor in the potency of the vaccine [5]. Thermostability is of particular importance due to the rugged conditions typically encountered in the regions of the world affected by infectious disease.

Previous work in our lab has shown that we have been able to improve on the typical viability and stability achieved through lyophilization. This is done by spray drying the bacteria in a dilute osmolyte solution. Increasing the osmolyte concentration in spray dried solution leads to less viability ultimately reflecting stresses that lead to cell death [6].

In general mycobacteria have well known cellular responses to environmental crisis and stresses such as heat shock, cold shock, nutrient limitation, and osmotic and oxidative stresses [7]. During the formulation process mycobacteria are exposed to stresses, which can cause cell damage and death. It is likely that bacteria that can survive the spray drying process more significantly express protective agents that render these bacteria more resistant to osmotic, heat and nutrient limitation stresses. We have therefore hypothesized that by repeatedly exposing bacteria to stresses involved in the processes of spray drying and dry state containment, we might succeed in selecting for bacteria populations with greater biochemical and biophysical ability to survive.

We chose to work with *M. smegmatis* as an illustrative mycobacterium given relative rapid growth and previous experience in spray drying. We spray dry *M. smegmatis* in dilute osmolyte conditions, recover the dry powder and expose the dry powder to 40 °C conditions sufficiently long enough to eliminate nearly all viable bacteria. We then re-suspend the highly stressed dry powder in culture media and grow the remaining live bacteria to stationary phase. This process was repeated (cycled) several times after which we examined the bacterial RNA through microarrays to quantify differences in gene expression.

By selecting viable bacteria in harsh stability conditions and identifying protective factors that allow them to survive, we hope to identify mechanisms through which highly robust and thermostable bacteria may be formulated so as to persist in the dry powder state. Ideally these results could then be applied to a broad range of live or attenuated whole-cell vaccines against infectious pathogens including M. *tuberculosis*.

## 2. Results and Discussion

*M. smegmatis* cultures were formulated into dry powders and placed in accelerated stability conditions at 40 °C and the viability was followed over time. The dry powders were prepared from: (1) bacteria growing in optimal exponential growth phase conditions (2) bacteria that had entered stationary phase and (3) bacteria that were exposed to repeated spray drying and post-drying exposure to 40 °C conditions – for four cycles of spray drying.

### 2.1. Viability

**Figure 1:**
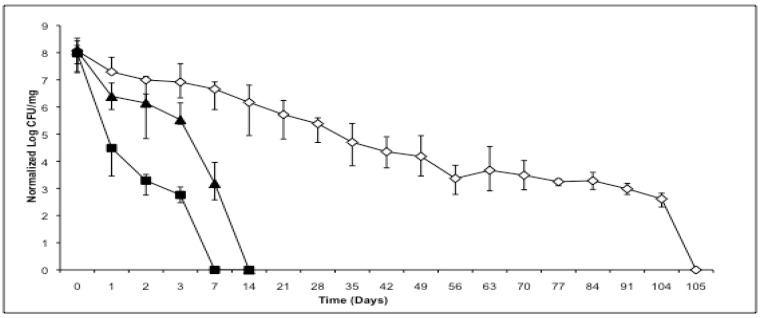
Viability comparison of dry powder *M. smegmatis* spray dried under various conditions. Normalized log CFU viability of *M. smegmatis* spray dried at log phase growth and stored at 40 °C (■). Normalized log CFU viability of *M. smegmatis* spray dried at stationary phase growth and stored at 40 °C (▲). Normalized log CFU viability of multiply cycled *M. smegmatis* stored at 40 °C (◇). Error bars represent maximum and minimum CFU at each time point across n. For cycled bacteria n = 3 was performed on the final (fourth) cycle.

As illustrated in Figure 1, bacteria dried after growing in log phase conditions exhibit the least resistance to the accelerated stability conditions, resulting in complete loss of viability within 7 days (n = 3). When the bacteria are grown to stationary phase for 24 hours, and then spray dried, they are able to survive longer in the desiccated state at accelerated stability conditions, with no detectable colonies after 14 days (n = 3). Viability over time in the desiccated state continued to increase as the formulations were cycled through the drying and heat-exposure process. “Cycling” consisted of repeated application of the following steps: first culturing bacteria to stationary phase, then processing cultures for spray drying (centrifugation and re-suspension in low osmolyte excipient solutions), then spray drying, then collecting and processing the dry powder (vial filling), then incubating the vials at 40 °C in stability chambers until viable bacteria were mostly eliminated, then culturing surviving bacteria from dry powder to stationary phase. After repeating the cycle four times (“multiply cycled bacteria”) the bacteria showed an almost 10-fold increase in stability with the ability to form colonies until 105 days (n = 3).

### 2.2. Phenotype

**Figure 2:**
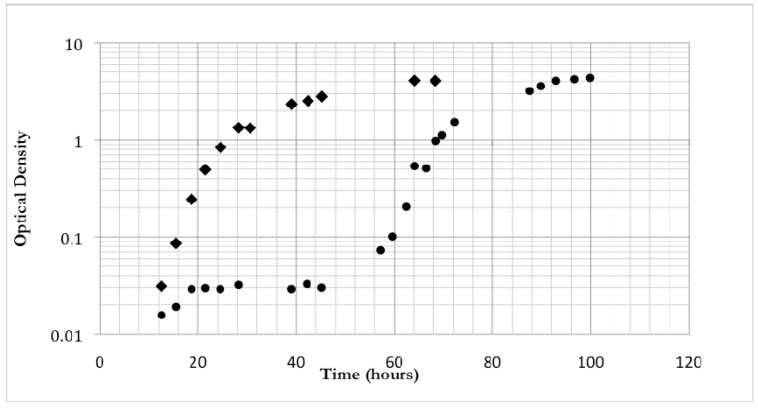
Optical density growth curves over time of non-spray dried *M. smegmatis* (◆) and multiply cycled *M. smegmatis* (●).

**Figure 3:**
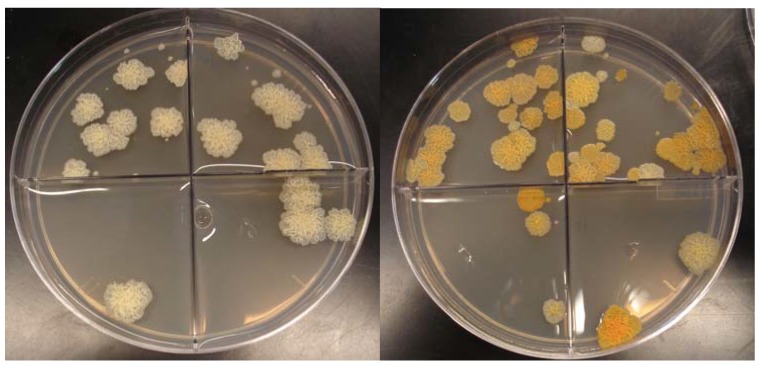
*M.* *smegmatis* colony forming units of (a) wild-type non-spray dried bacteria and (b) multiply-cycled bacteria after 1 day exposure to light and air. Bacteria are not exposed to light during incubation. The orange phenotype will emerge in the wild-type strain after exposure to air and light at low frequency. Multiply-cycled bacteria emerge from the incubator with the orange phenotype which becomes more intense upon exposure to light and air.

The increased viability over time of multiply cycled bacteria was accompanied by some minor changes in growth rate and overall gross morphology differences between the colony forming units. In log growth phases, the wild type non-spray dried bacteria exhibited a doubling time of 2.4 ± 0.3 hours (n = 3), whereas multiply cycled bacteria doubled approximately every 3.1 ± 0.1 hours (n = 3) (Figure 2). Surface topology was identical between colonies with both the non-previously spray dried bacteria and the multiply cycled bacteria exhibiting rough morphology. Strikingly, the color of the multiply cycled bacteria colonies differed from the non-spray dried bacteria. Approximately 30 ± 5% of the colonies on multiply cycled plates were orange pigmented upon removal from the plate incubator whereas only 5 ± 3% of the wild type non-spray dried plates were orange colored upon removal. This pigmented phenotype began to emerge after the second spray drying cycle and became dominant by the fourth cycle. The proportion of multiply cycled colonies exhibiting pigmentation, as well as the intensity of the pigmentation, increased when plates were left on the bench-top and exposed to light and air. The percentage of heavily pigmented colonies grew to greater than 90% ± 5% after 1 day exposure to light and air (Figure 3).

### 2.3. Gene Expression

We performed two sets of gene expression experiments to uncover factors important for sustained viability in the dry powder formulation process. In the first experiment we examined gene expression in log phase and stationary phase cultures of bacteria, neither of which had been previously exposed to spray drying. In our second set of experiments we compared gene expression in non-previously spray dried bacteria to that in multiply spray dried bacteria. In this case we made head-to-head comparisons in log phase, stationary phase, and dry powders that had 24 hour exposure to accelerated stress conditions.

#### 2.3.1. Log *versus* Stationary Comparison in Non-Spray Dried Cultures

We extracted RNA from log phase (O.D. = 1.0) and stationary phase (O.D. > 3.0) bacteria and performed four microarrays - two biological replicates each with a dye swap to minimize dye specific bias. As expected, significant differential gene expression was observed. Out of approximately 7000 genes on the microarray, about 2500 were differentially expressed at a p-value < 0.05 level of significance. Out of these 2500 genes, approximately 1400 were differentially expressed with a p-value < 0.01. The log 2 median average intensity of the *M. smegmatis* spots was 9.8 whereas the median average intensity for the A. thaliana control spots was 7.2. This indicated that signal was, on average, 5-fold greater than non-specific cross-hybridization noise.

Genes up-regulated in log phase over stationary phase included a nearly complete complement of ribosomal proteins (Appendix Table 1) as well genes that are important for growth including electron transport (e.g. ATP synthase components), energy metabolism (e.g. TCA cycle enzymes), and cell maintenance needs (e.g. lipid metabolism and protein folding) (Appendix Table 2). Genes up-regulated in stationary phase over log phase included those typically associated with states of stress including catalases, nitrite reductases, alternative sigma factors, and various amino acid permeases and transporters (Appendix Table 3). Two clusters related to the expression and assembly of [NiFe] hydrogenase were up-regulated along with other stress related genes included UsfY (MSMEG_1769 and MSMEG_1791), the starvation-induced DNA protecting protein (MSMEG_6467), the sporulation factor WhiB (MSMEG_1597 and MSMEG_1953), and L-lysine-epsilon aminotransferase (MSMEG_1764).

Since differential regulation of gene expression is mainly controlled by the presence of primary and alternative sigma factors we expected to see significant up-regulation of MysA (primary housekeeping factor) in log phase and sigB and sigF in stationary phase (stress related factors) [8]. While we found that these were three of the six most highly expressed transcripts, as measured by average intensity across all channels, there was little evidence of differential expression (Appendix Table 4). Instead we found that two sigma factors related to the sigma-54 factor (nitrogen limitation and alternative carbon utilization [9]) and two sigD factors (alternative stress [10]) were most differentially expressed with respect to stationary phase as well as a large (100kD), uncharacterized sigma factor expressed with respect to log phase.

#### 2.3.2. Non-Previously Spray Dried *versus* Cycled

In our second set of experiments, we performed microarray analysis that compared gene expression in bacteria that had never been spray dried to that in bacteria that had been subjected to multiple spray drying cycles. We compared the differently processed bacteria by performing four microarrays in log phase (two biological replicates each with a dye swap), three microarrays in stationary phase (two biological replicates with a single swap), and two microarrays in dry powder form (single biological sample with a dye swap). In the log phase comparison, 79 genes were differentially expressed with a p-value < 0.05 of which 36 were differentially expressed at a p-value < 0.01 level of significance. All but two of these genes, acyl-CoA dehrydrogenase (MSMEG_1821) and malonyl CoA-acyl carrier protein transacylase (MSMEG_4325), were upregulated in the multiply cycled bacteria.

In the stationary phase comparison there were no genes differentially expressed at p-value < 0.05 level of significance. However, using the log odds scores calculated by the Limma statistical package we found that there were ten genes that had 50% or greater probability of differential expression (three up-regulated in non-cycled bacteria and seven up-regulated in multiply cycled bacteria – see Appendix Table 6). In addition, there was a significant number that had some (>10%) probability of differential expression. In the dry powder comparison there was a much higher level of differential expression. Approximately 1200 genes were differentially expressed with p-value < 0.05, however, of these only 140 had a p-value < 0.01 and the number of genes that had a 50% or greater chance of being differentially expressed was only 291. The median average intensity for the *M. smegmatis* spots in this comparison was 8.3, approximately 2-fold below the medians for both the log phase (9.1) and the stationary phase (9.5) comparisons indicating a lower level of signal.

#### 2.3.3. Log Phase Comparison

Results for the log phase differential expression data are given in Appendix Table 5. The differentially expressed genes are dominated by a large gene cluster (22% of the statistically significant genes) that runs from MSMEG_1766 to MSMEG_1802. Two copies of the UsfY gene product (MSMEG_1769; MSMEG_1777) in the cluster are differentially expressed whereas a third copy of UsfY in the cluster (MSMEG_1791), the one that is closest upstream to sigF and most highly expressed in stationary phase, is not differentially expressed. A fourth copy of UsfY (MSMEG_4406) elsewhere in the genome is also not expressed. SigF is likely expressed, based on an intensity 1.2 standard deviations above the median average intensity, but not differentially (intensity ratio = 0.1). S-(hydroxymethyl) glutathione dehydrogenase is differentially expressed at two loci (MSMEG_0671; MSMEG_6616). Also differentially expressed were genes involved in the acquisition or production of osmolytes and carotenoid antioxidants (e.g. MSMEG_2926 and MSMEG_3184; MSMEG_2345 and MSMEG_2346), two catalases (MSMEG_6213; MSMEG_6232), and the starvation-induced DNA protecting protein (MSMEG_6467).

#### 2.3.4. Stationary Phase Comparison

Stationary phase microarray data did not have any statistically significant differentially expressed genes. However, many transcripts did have positive probability of differential expression (Appendix Table 6) with phytoene synthase (MSMEG_2346) having the highest probability of differential expression (66%). Other differentially expressed transcripts include phytoene dehydrogenase (MSMEG_2347), which participates in the same biosynthetic pathway as phytoene synthase, a manganese containing catalase (MSMEG_6213), maltooligosyl trehalose synthase (MSMEG_3185), S-(hydroxymethyl) glutathione dehydrogenase (MSMEG_0671), and the MSMEG_1769 locus of UsfY. Genes appearing in the stationary phase comparison but not in the log phase comparison include glycerol kinase (MSMEG_6759), glycerol-3-phosphate dehydrogenase 2 (MSMEG_6761), and AmiB (MSMEG_1679). Notably, these three genes were down-regulated relative to the cycled bacteria. SigB (MSMEG_2752) was up-regulated in this comparison where it was not observed to be differentially expressed in the previous non-previously spray dried log *versus* stationary phase experiments.

#### 2.3.5. Dry Powder Comparison

The dry powder comparison showed that the non-cycled bacteria increased transcriptional expression of genes associated with growth processes (Appendix Table 7). These transcripts included those for glycolysis (MSMEG_4107), sulfur uptake (MSMEG_5789), fatty acid metabolism (MSMEG_2081; MSMEG_6512), and amino-acid biosynthesis (MSMEG_1843). In addition, there were expressed transcripts related to shut-down or repair including those for amino acid scavenging (MSMEG_5486; MSMEG_6332), oxidative damage (MSMEG_3215), nucleic acid degradation (MSMEG_3902; MSMEG_5226), and the soluble pyridine nucleotide transhydrogenase (MSMEG_2748), which catalyzes the conversion of NADH to NADPH and is important for catabolic processes. Genes expressed at higher levels in cycled bacteria contained a number of genes related to lipid synthesis, a diverse group of transposable elements, the stress related sigD alternative sigma factor (MSMEG_1599), and the error-prone DNA polymerase IV (MSMEG_2748) (Appendix Table 8).

### 2.4. Viability Discussion

The results of this study show that the processing of bacteria into a dry powder state affects overall fitness and ultimately survivability. It is important that fitness, or the ability to respond appropriately to specific stress conditions, not require processing conditions that inhibit the bacteria’s ability to flourish in normal growth or other environments. In this light it is important that the bacteria show improved viability over time when grown to stationary phase and exposed multiple times to accelerated stability conditions and the spray drying process. Although the cycled *M. smegmatis* doubles at a slightly slower rate, 3.12 hours *vs.* 2.36 hours, both times are well within the literature reported values of the bacteria’s doubling time under normal growing conditions [11,12]. Furthermore, we found little evidence in the gene expression data to suggest that the observed variability in growth rate was related to transcriptional differences. There was no differential expression observed in genes central to growth or maintenance and limited differential expression overall. However, the genes that were differentially expressed were heavily skewed in number towards the cycled bacteria. The additional expression in cycled bacteria could represent a small increased energy demand in which case the observed slower metabolism might be a genuine consequence of our formulation process.

### 2.5. Gene Expression Differences

Our expression data illustrate that the transition to growth phase from stationary phase is a smooth and highly orchestrated switch in metabolic profile. Stationary phase is a natural response to stressful conditions and bacteria have robust systems in place to counter environmental challenges. In stationary phase of both non-cycled and cycled bacteria we observed increased expression of products that are used to fight stress. These products (Appendix Table 3) included those that combat reactive oxygen species [13], compensate for nitrogen limitation [14], facilitate the utilization of alternative carbon sources [15], and provide for metabolic scavenging [16]. The upregulation of these [NiFe] hydrogenase related genes suggests a response to oxygen limitation ([NiFe] hydrogenases have been shown to be strongly upregulated in hypoxic conditions [17]). Intriguingly, L-lysine-epsilon aminotransferase has been shown to be 40-fold up-regulated in models of the persistent/latent infection of M. tuberculosis [18]. It is probable then that the observed increase in dry powder viability of stationary phase cultures over log phase cultures is a consequence of bacteria being better suited to resist harsh conditions.

In a similar vein, our data suggest that in repeatedly stressing bacteria we have enriched the capacities by which bacteria can survive new and specific stress conditions. Interestingly, these capacities seem to be manifested such that the cycled bacteria “anticipate” future stress. For example, the over-production of trehalose biosynthetic enzymes (trehalose is an excellent osmoprotectant), catalases (to neutralize reactive oxygen species), and glutathiones (for alternative carbon utilization and antioxidant activity) occurs in both log and stationary phases of cycled bacteria. Glycerol kinase and glycerol-3-phosphate dehydrogenase 2 are both down-regulated in stationary phase in cycled bacteria. Since both of these enzymes are involved in processing of glycerol, the down-regulation of these two enzymes has the likely effect of increasing intracellular glycerol concentrations. Given that glycerol is another highly effective osmoprotectant (and water substitute), accumulation undoubtedly helps protect against the osmotic forces at work in the drying process and in the dry powder state. Likewise, AmiB, which plays a role in maintenance and disassembly of the extra-cellular polysaccharide capsid, is also down-regulated in stationary phase in cycled bacteria. It may make “survival-sense” for bacteria to reduce degradation of an all important cell barrier if stress is on the horizon. Moreover, a very interesting result was that of the starvation-induced DNA protecting enzyme which is over-produced beginning in log phase growth. This protein is known to exist in two multimeric forms with the extended polymeric form conferring the principle protection of DNA [19]. The transition from the limited multimeric form to the extended polymeric form is temperature dependent, occurring at 40 °C. Since our spray drying was carried out at +40°C and powders subsequently incubated at 40 °C for extended periods of time, it is possible to speculate that the observed increase in expression is a direct response to our processing conditions. That is, since there is a significant amount of DNA to protect in the event of heat stress, and our processing occurs rapidly, it clearly benefits the organism to accumulate this protein preemptively.

### 2.6. Carotenoids

One striking observation in our study was the marked orange color and continued rapid orange transformation of the cycled bacteria. It was observed however that a fraction of colonies from wild type cultures would also undergo a similar color transformation. It is known that stock cultures of *M.* *smegmatis* often contain pigmented colonies (as well as other variants) suggesting multiple sub-populations exist or arise naturally in the mc^2^155 strain [20]. In our case this phenotype emerged dominantly when large populations were repeatedly spray dried and placed in the stressful environment of a heated dry powder suggesting the orange phenotype may be related to a selective advantage.

Carotenoids are a class of isoprenoid metabolites synthesized de novo in bacteria. The carotenoid pathway ultimately results in pigmented complex polyterpene lipids including-carotene and lycopene whose functions are in part to act as free radical scavengers and protect cells from light induced oxygen species [21]. The carotenoids are also known to be able to contribute to enhancing the strength of the cell wall due to their lipophilic nature and intercalation into the cell membrane [22]. The presence of gene products that catalyze the formation of these compounds almost certainly explains the pigmentation appearing in the multiply cycle bacteria including the observed increase in color intensity when exposed to light and dry air on the benchtop. Since carotenoids are robust antioxidants and fortifiers of cellular barriers they would be beneficial for withstanding the shear and osmotic stress in the dry powder formulation procedure. In fact, the buff colored mc^2^155 strain of *M. smegmatis* is known to be less robust relative to the naturally pigmented wild-type strains, having seen ongoing usage as a model organism, in part, for its high transformation efficiency [23,24]. Thus, we feel the putative over-production of these compounds in cycled bacteria would support our hypothesis that pre-stressed bacteria are more robust.

Analysis of the microarray data showed that the entire carotenoid biosynthetic operon is up-regulated in the cycled bacteria in both log and stationary phases (Appendix Table 9). We note that the pathway is not differentially expressed in the dry powder state, however, the high signal intensity over both the cycled and non-cycled samples (all five genes in the operon had expression levels two standard deviations or higher than the median expression level) suggests that it is highly expressed in both cases.

**Figure 4:**
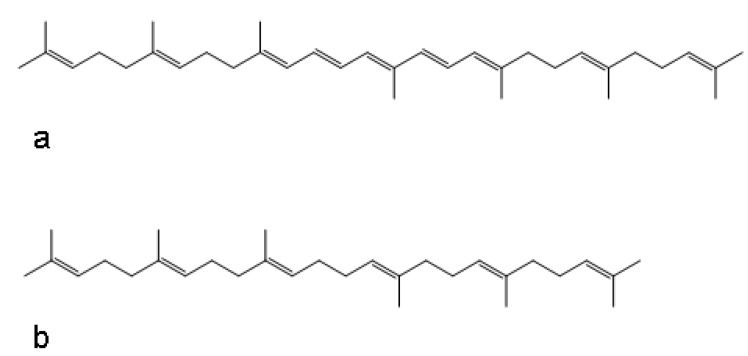
Structures of (a) zeta-carotene and (b) squalene.

Importantly, previous work conducted in our lab investigated the effects of adding the commercial adjuvants titermax and titermax gold in attempts to increase immunity and antigenicity in spray dried bacteria. It turns out that the major component of the commercial adjuvant formulations are squalene derivatives. These structures have highly similar structure properties with the naturally occurring mycobacteria carotenoids such as zeta-carotene (Figure 4). Remarkably these adjuvant/bacteria formulations also showed a 1–5 log improvement in viability over time in the dried powder state (unpublished data). This suggests that cartenoid and squalene derivatives may play a critical role in increasing viability of organisms in formulation processes and in the dry powder state over time.

### 2.7. Stress Response Gene Cluster

The observation that the gene cluster [MSMEG_1750 to MSMEG_1804] is up-regulated in cycled bacteria is a significant observation. Several genes in this cluster are thought to be related to or regulated by the alternative sigma factors sigF and sigD, including three copies of UsfY (upstream of sigma F protein Y). This cluster of genes is highly similar to a cluster of stress related genes (also containing UsfY) that is implicated in the latency and persistence of M. *tuberculosis* [25,26].

It has been postulated that UsfY is an anti-anti-sigma factor directed at sigF [25]. Sigma factors act as critical regulators of gene expression in bacteria by recognizing their cognate promoters and controlling the different programs that bacteria employ in response to environmental stimuli. Anti-sigma factors bind to sigma factors to down-regulating specific transcriptional activity. In turn anti-anti-sigma factors bind to anti-sigma factors and thus dampen their regulatory activity. Thus, up-regulation of UsfY would help explain the increased levels of sigF-dependent transcripts in stressed bacteria.

It has been shown that carotenoid biosythesis genes are regulated by sigF in *M.* *smegmatis* [21]. Given the high level of gene expression we observed in the carotenoid biosynthetic pathway, as well as in the cluster of genes related to sigF, we did a simple promoter search in the *M. smegmatis* genome for the sigF consensus promoter sequence -10 (GGGTTT) [26]. The results were striking. A large number of genes that were seen to be either differentially expressed in the cycled bacteria (log and/or stationary phase), or highly expressed in the dry powder state, appear to be directly regulated by sigF (Appendix Table 10). In addition, it appears that the MSMEG_1777 locus of UsfY is itself regulated by sigF. Since sigF itself was not seen to be differentially expressed in any of the experiments, including the non-spray dried log *versus* stationary phase comparison, higher levels of sigF controlled products in the cycled bacteria was puzzling. One possibility is that higher levels of these products could have arisen from increased UsfY expression, at other locus not under control of sigF, combined with basal sigF expression. This, by itself, might account for the observed improvement in viability of cycled bacteria. However, the high expression levels and postulated anti-anti-sigF activity of UsfY, along with the positive regulation by sigF (at least at the MSMEG_1777 locus) may provide for a mechanism by which the cycled bacteria produce larger quantities of important products in a just-in-time manner, thus conserving resources while simultaneously being prepared to better survive the dry powder formulation. The mechanism may be that UsfY acts like a positive gain in a control circuit. That is, since UsfY is positively regulated by sigF, higher levels of sigF lead to higher the levels of UsfY, and because of the anti-anti sigma factor activity, higher levels of UsfY lead to higher activity of sigF and consequently higher levels of stress related products (e.g. carotenoids). This feedback control, along with the coordinated anti-sigma factor activity, is a well established regulation mechanism for transcriptional control used in bacteria. However, our results suggest that the multiply cycled bacteria may constitutively express higher levels of UsfY and by doing so they likely introduce positive gain into the system. At higher initial levels, UsfY is positioned to shift the equilibrium away from anti-sigF factors as they are produced. Stress signals that increase sigF levels (such as drying stress) would be rapidly amplified since any concomitantly produced anti-sigF factors would be immediately sequestered. In this way, multiply cycled bacteria can not only respond more robustly to stress stimuli but also faster. We feel the latter is an exceedingly important point as our spray drying procedure imposes an extreme change in environment over a very short timeframe.

The lack of differential expression of the UsfY cluster of genes in the dry powder further supports the idea that increased expression is more beneficial *prior* to the actual drying phase. In other words, strengthening of the cell wall, or accumulating a pool of antioxidants, or preparing for osmotic stresses, is best done proactively because once in the dry powder state energy may be required for other processes (such as repair). This postulate is evidenced by the overall transcriptional responses in dry powder. In the absence of “preparative” gene expression, the non-cycled bacteria appear to have increased expression of genes related to basic metabolic needs. This could reflect a slightly heightened response to the nutrient limited conditions, a last ditch effort to produce energy and acquire necessary components for maintenance, or an attempt at repair. In any case, the increased expression of these products appears to be insufficient (based on differences in viability) and too limited given the extreme urgency needed in adaptation to the harsh and resource-poor environment. In contrast, genes up-regulated in the cycled bacteria suggest an attempt to cope with extreme stress with extreme measures. The increased expression of error-prone DNA polymerase IV, which provides a mechanism for adaptive mutagenesis, suggests this is the case while the number of transposases expressed indicates that dry powder environment is, in fact, catastrophic for the bacteria. Transposases facilitate the “jumping” of DNA segments randomly across genome in an effort to form new recombinant proteins to help combat a new stress. In our data we see that the IS1096 transposable element is highly and differentially expressed in cycled bacteria in the dry powder state. In addition, IS096 related transcripts (Appendix Table 8) include hypothetical proteins that have the IS1096 transposon partially overlapping on the complimentary strand. Transposons are known to contain complimentarily coded regulatory sequences (*i.e.* sigma factor binding sites) and the fact that these hypothetical proteins are being expressed in the dry powder state makes it highly likely that transposon mediated mutagenesis is in fact occurring. Our promoter analysis identified at least one copy of the IS096 TnpR transcript (MSMEG_4791) as being regulated by sigF and thus higher expression of IS096 in cycled bacteria is consistent with the cycled bacteria’s UsfY augmented sigF response. Thus, “preparative” expression in cycled bacteria may be conferring an adaptive advantage in that an organism that can devote more energy and cellular resources to recombination, over one that has to scavenge more resources for maintenance and repair, has a substantially higher probability of surviving extreme duress.

In summary, our data suggests that the acquisition of enhanced cartenoid synthesis enhances post spray‐drying dry powder viability. This enhanced synthesis could potentially result from a mutation in sigF or possibly from IS1096 transposition into regulatory sequences. Further work will be required to determine if the multiply spray dried phenotype, which we have designated MSDsigf(+) (Table 1), and the high cartenoid phenotypes share a common mutation. In particular, sequencing of the sigF region of the chromosome will be of high priority.

**Table 1:** Strain Table.

Strain Number	Description	Origin
mc^2^155	High-frequency transformation mutant of ATCC 607	Bloom lab. Snapper *et al* [27]
MSDsigf(+)	Putative sigF regulatory mutant, with high carotenoid content, isolated from multiply spray dried powders.	Edwards Lab

## 3. Experimental Section

### 3.1. Culture and Growth Conditions

*M.* *smegmatis* mc^2^155 was generously provided by Dr. Barry Bloom of the Harvard School of Public Health. Mc^2^155 was cultured in standard minimal media, Middlebrook 7H9 with 10% OADC (BD Diagnostics, Rockville, MD), 0.2% glycerol (Sigma, St Louis, MO), and 0.05% Tween 80 (Sigma), supplemented with 50 μg/mL hygromycin (Roche, Indianapolis, IN) and incubated at 37 °C. Late exponential phase cultures were grown to an optical density (O.D.) of 1.3 (~24 hrs). Stationary phase cultures were grown for three days to an O.D. > 3.0.

### 3.2. Solution Preparation

Spray drying solutions were prepared by pelleting cultures, washing them with PBS/0.05% Tween 80, and resuspending them in an equal volume of 0.05% Tyloxapol (Sigma). The final solution was mixed with an equal volume of 8 mg/mL L-leucine (Sigma) for a final concentration of 4 mg/mL L-leucine and 0.025% Tyloxapol. All solutions were used immediately after preparation.

### 3.3. Spray Drying Conditions

Spray drying was carried out in a Buchi B-290 mini spray dryer using a high performance cyclone and a 0.7 mm pressure nozzle tip (Buchi, Flawil, Switzerland). Solutions were spray dried at a feed rate of 7 mL/min with a drying air flow rate of 35 liters/hr. Outlet temperature was kept between 42–45 °C by varying the inlet temperature from 115–125 °C. The day-to-day variation was due to differences in ambient relative humidity. Powder was collected immediately and placed into amber scintillation vials. The vials were then stored in a desiccator placed in either a 40 °C/ 75% or 25 °C/ 60% relative humidity chamber.

### 3.4. Viability

Serial dilution plating followed by CFU determination was used to assess the number of viable *M.* *smegmatis* bacteria in cell suspensions before spray drying and in the powders post spray drying. Briefly, powders were resuspended in PBS/0.05% Tween 80 and vortexed to homogeneously disperse the samples. Samples were then serially diluted and placed on Middlebrook 7H10 agarose with 10% OADC, 0.5% glycerol and supplemented with 50 μg/mL hygromycin. Plates, once inoculated, were wrapped in foil and incubated at 37 °C for three days. In order to assess the stability of the bacteria over time, powders were placed in storage conditions and plated at regular intervals.

### 3.5. RNA Extraction

RNA was extracted from either 25 mL of culture or 200 mg of powder. Powder was first resuspended in 25 mL of DEPC water (Ambion, Austin, TX). Both solutions were then pelleted by centrifuging at 10,000 rpm for 1 min. Extraction was then carried out as described in Managan *et al*. [28]. Briefly, 0.4 mL DEPC H_2_O and 1 mL of detergent solution (Tween-80, SDS (Sigma), 0.5M Sodium Acetate, DEPC H_2_O) were added to the pellet and gently mixed. The mixture was added to 4 mL of 0.1 mm silica/ceramic beads in a 7mL screw-top beadbeater tubes. Phenol: chloroform: isoamyl alcohol 125:24:1 (Sigma) and chloroform: isoamyl alcohol 24:1 (Sigma) were then added to the tubes. The mixture was bead beat on high for 45 sec on a Biospec Mini-Bead Beater™. The broken cells were placed on ice for 10 min. The liquid was transferred to 2 mL screw-capped tubes and centrifuged at 16 X rpm for 10 min. The aqueous phase was removed and transferred to a fresh 2 mL screw-cap tube, equal volume chloroform isoamyl alcohol was then added. The solution was briefly centrifuged and the aqueous phase was once more removed. An equal volume of isopropanol solution was then added. Tubes were placed in −80 °C freezer overnight. The tubes were centrifuged at 16 X rpm for 15 min, the supernatant was poured off, and the pellet dried for 45 min on the bench top. RNA cleanup was carried out using a Qiagen RNeasy® Mini Kit with DNAse digestion. Total RNA was eluted in 60 μL and concentration was determined on a NanoDrop ND-1000. RNA content was visually verified by running samples on precast agarose gels (Sigma) in a mini gel electrophoresis unit with ethidium bromide staining.

### 3.6. cDNA Synthesis and Aminoallyl-labeling

cDNA was synthesized by adding 2 μg of total RNA to 2 μL of random hexamers (Invitrogen, Grand Island, NY) and nuclease free water (Ambion) to achieve a final volume of 18.5 μL. Samples were incubated at 70 °C for 10 minutes, snap-frozen on ice and then centrifuged at 10,000 rpm. The solution was then added to 6 μL first strand buffer (5X) (Invitrogen), 3 μL 0.1 M DTT, 0.6 μL 25 mM dNTP/aa-UTP labeling mix, and 2 μL PowerScript RT (Invitrogen). The mixture was then incubated in a 42 °C water bath overnight. RNA was hydrolyzed by adding 10 μL 0.5 M EDTA (Ambion), 10 μL 1 M NaOH, and then incubating at 65 °C for 15 minutes. Next, 25 μL 1 M TRIS (pH 7.0) (Ambion) was added in order to neutralize the pH. Unincorporated aa-DUTP and free amines were removed with a Qiagen MiniElute PCR purification kit. cDNA was eluted in 60 μL and the concentration determined on a NanoDrop ND-1000. The cDNA was then dried in a speed vac. Samples were resuspended in 4.5 μL 0.1 M sodium carbonate buffer pH 9.3 and added to 4.5 μL of either Cy3 or Cy5 dye (Amersham). The solutions were allowed to incubate in the dark at room temperature for 1 hour. After coupling had finished, 35 μL of 100 mM NaOAc pH 5.2 was added and the samples were purified using a Qiagen MiniElute PCR purification kit used according to the manufacturer instructions. Dye incorporation was assessed using the NanoDrop ND-1000 microarray analysis settings.

### 3.7. Microarray Preparation and Hybridization

*M.* *smegmatis* microarrays were generously provided by The Institute for Genomic Research (TIGR). Hybridization of labeled cDNA probes was carried out using the TIGR SOP M007/8. Briefly, microarray slides were incubated in a prehybridization solution at 42 °C in coplin jars for 1 hour. Slides were then transferred to a glass staining dish and washed 10X with 200 mL nuclease free water. The slides were then rinsed for 2 min in a staining dish filled with isopropyl alcohol and then centrifuged at 1000 rpm for 10 min to dry. A 40% formamide hybridization buffer was then prepared and 50 μL was added to the cy3/cy5 probe. The probe mixture was placed on a 95 °C heat block for 5 min, vortexed and then heated for another 5 min. Prehybridized microarray slides were placed in a hybridization chamber with a clean LifterSlip (Erie Scientific, MA) and the probe mixture was added. A small amount of unused hybridization solution was added to each of the small wells located at either end of the microarray slide. The chamber was wrapped in foil and incubated in a 42 °C water bath overnight. After hybridization, slides were sequentially washed in 500 mL low stringency, medium stringency and high stringency buffers. Each wash step was carried out twice in glass staining dishes. Slides were rinsed briefly in 500 mL Millipore water and centrifuged for 2 min at 1000 rpm and scanned.

### 3.8. Image Scanning and Data Analysis

Microarrays were scanned using an Axon scanner and data was acquired using Genepix Pro 5.1.0.19 software. Data was analyzed using Bioconductor bioinformatic software with the Limma statistical package [29]. Data was filtered to exclude poor spots (Flag > −50). Background was corrected using the backgroundCorrect command and data was normalized using the normalizeWithinArrays command. Adjusted data was then fit to linear and Bayesian models using the lmFit and eBayes commands. Intensity Ratios, Average Median Intensity and p-values were taken from the logFC, AveExpr, and the more stringent Adj. P.Val. in the output file and then averaged over the three gene replicates present on each microarray. Probability of Differential Expression was calculated using the Limma log-odds score (B) and equation (1).

(1) $$\left(\frac{e^{\left(B\right)}}{1+e^{\left(B\right)}}\right)\times 100$$

## 4. Conclusions

Our results suggest that relevant stressing of bacteria, such as M. *smegmatis*, can lead to highly stable dry powder formulations with remarkable room temperature stability characteristics. Repeated spray drying and selective pressures in dry powders may enrich for strains which can persist in harsh conditions. It is likely we have selected a natural population most fit for long term survival in dry powders which in theory could make for more stable vaccines. However, it is clear that the dry powder state is exceedingly harsh and may induce recombination events. In applying our methodology to more relevant vaccine strains it will be important to ensure they retain immunogenicity and remain safe.

We have demonstrated a new approach useful in the formulation of live whole-cell vaccines. This approach centers on the biochemistry of the organism rather than the chemical and physical parameters often the focus of vaccine formulation efforts. The approach has not only provided insight into mechanisms that influence viability, but has also led us to specific compounds that may prove advantageous in the dry powder formulation of other important organisms.

## Appendix Tables–Microarray Data

**Appendix Table 1:** Ribosomal genes up-regulated in log phase (non-spray dried log *versus* stationary comparison).

Primary Target	Common Name	Gene	Intensity Ratio	Average Channel Intensity	p-value	Probability of Differential Expression
MSMEG_1347	ribosomal protein L1	rplA	1.4	11.8	0.001	88%
MSMEG_1439	ribosomal protein L2	rplB	1.3	12.6	0.001	81%
MSMEG_1436	ribosomal protein L3	rplC	1.2	11.4	0.001	97%
MSMEG_1437	ribosomal protein L4/L1 family protein	rplD	1.3	12.1	0.000	99%
MSMEG_1467	ribosomal protein L5		1.8	11.8	0.000	100%
MSMEG_1470	ribosomal protein L6		1.2	12.6	0.001	94%
MSMEG_6894	ribosomal protein L9	rplI	1.0	11.3	0.001	89%
MSMEG_1364	ribosomal protein L10		2.1	12.7	0.000	99%
MSMEG_1346	ribosomal protein L11	rplK	1.5	13.1	0.000	99%
MSMEG_1365	ribosomal protein L7/L12	rplL	2.8	11.9	0.001	95%
MSMEG_1556	ribosomal protein L13	rplM	1.8	13.5	0.002	65%
MSMEG_1465	ribosomal protein L14	rplN	1.1	11.3	0.004	55%
MSMEG_1474	ribosomal protein L15	rplO	1.3	11.8	0.002	77%
MSMEG_1443	ribosomal protein L16	rplP	1.8	13.0	0.001	95%
MSMEG_1525	ribosomal protein L17		1.1	12.3	0.003	59%
MSMEG_1471	ribosomal protein L18	rplR	1.6	12.2	0.000	98%
MSMEG_2440	ribosomal protein L19	rplS	1.2	11.4	0.001	92%
MSMEG_3791	ribosomal protein L20	rplT	0.6	12.1	0.010	17%
MSMEG_4625	ribosomal protein L21	rplU	1.3	12.9	0.001	96%
MSMEG_1441	ribosomal protein L22		1.5	11.1	0.006	33%
MSMEG_1438	ribosomal protein L23	rplW	1.4	12.1	0.000	98%
MSMEG_1466	ribosomal protein L24	rplX	1.1	12.0	0.001	96%
MSMEG_5431	ribosomal protein L25, Ctc-form		1.9	11.3	0.000	99%
MSMEG_4624	ribosomal protein L27	rpmA	0.2	12.9	0.324	0%
MSMEG_6068	ribosomal protein L28	rpmB	0.1	9.8	0.734	0%
MSMEG_2400	ribosomal protein L28	rpmB	1.5	12.8	0.000	99%
MSMEG_1444	ribosomal protein L29	rpmC	1.8	11.8	0.013	14%
MSMEG_1473	ribosomal protein L30	rpmD	1.5	11.9	0.001	95%
MSMEG_4951	ribosomal protein L31	rpmE	0.8	13.8	0.005	37%
MSMEG_5489	ribosomal protein L32	rpmF	1.1	11.1	0.010	22%
MSMEG_6070	ribosomal protein L31	rpmE	0.0	10.1	0.704	0%
MSMEG_6067	ribosomal protein L33	rpmG	-0.4	9.4	0.368	0%
MSMEG_1339	ribosomal protein L33	rpmG	0.6	11.6	0.045	9%
MSMEG_6946	ribosomal protein L34	rpmH	0.3	11.5	0.282	0%
MSMEG_3792	ribosomal protein L35	rpmI	0.8	13.6	0.004	46%
MSMEG_1520	ribosomal protein L36	rpmJ	0.4	13.3	0.080	1%
MSMEG_3833	ribosomal protein S1		-0.3	12.1	0.174	1%
MSMEG_2519	ribosomal protein S2	rpsB	2.0	11.7	0.000	100%
MSMEG_1442	ribosomal protein S3	rpsC	1.8	11.8	0.000	99%
MSMEG_1523	ribosomal protein S4	rpsD	1.0	12.0	0.002	68%
MSMEG_1472	ribosomal protein S5	rpsE	1.4	12.2	0.001	93%
MSMEG_6897	ribosomal protein S6	rpsF	2.9	11.6	0.000	100%
MSMEG_1399	ribosomal protein S7	rpsG	1.9	12.7	0.000	100%
MSMEG_1469	ribosomal protein S8	rpsH	1.8	12.4	0.001	93%
MSMEG_1557	ribosomal protein S9	rpsI	1.1	12.7	0.001	90%
MSMEG_1435	ribosomal protein S10	rpsJ	1.1	12.3	0.001	88%
MSMEG_1522	ribosomal protein S11	rpsK	1.2	12.8	0.001	88%
MSMEG_1398	ribosomal protein S12	rpsL	1.4	12.4	0.001	97%
MSMEG_6066	ribosomal protein S14		-0.6	9.0	0.255	0%
MSMEG_1468	ribosomal protein S14p/S29e	rpsN	1.6	12.1	0.003	60%
MSMEG_1521	ribosomal protein S13p/S18e	rpsM	0.9	12.3	0.014	12%
MSMEG_2654	ribosomal protein S15	rpsO	1.3	11.4	0.001	92%
MSMEG_2435	ribosomal protein S16		1.8	11.1	0.003	68%
MSMEG_1445	ribosomal protein S17		2.0	12.2	0.001	91%
MSMEG_6065	ribosomal protein S18	rpsR	-0.3	9.3	0.587	0%
MSMEG_6895	ribosomal protein S18	rpsR	1.4	12.2	0.001	88%
MSMEG_1440	ribosomal protein S19	rpsS	1.4	12.1	0.001	97%
MSMEG_4571	ribosomal protein S20	rpsT	0.5	12.1	0.033	4%

**Appendix Table 2:** Select up-regulated operons and clusters in log phase over stationary phase (non-spray dried log *versus* stationary comparision).

Primary Target		Common Name	Gene	Intensity Ratio	Average Channel Intensity	p-value	Probability of Differential Expression
**Electron transport**							
MSMEG_4939		ATP synthase delta chain		1.5	11.9	0.00	98%
MSMEG_4942		ATP synthase F0, A subunit	atpB	1.3	11.5	0.00	88%
MSMEG_4941		ATP synthase F0, C subunit	atpE	1.8	13.3	0.00	99%
MSMEG_4938		ATP synthase F1, alpha subunit	atpA	1.5	11.7	0.00	99%
MSMEG_4936		ATP synthase F1, beta subunit	atpD	1.5	12	0.00	99%
MSMEG_4935		ATP synthase F1, epsilon subunit	atpC	0.7	10.9	0.03	5%
MSMEG_4937		ATP synthase F1, gamma subunit	atpG	1.7	11.4	0.00	100%
MSMEG_4268		cytochrome c oxidase subunit 2		1.7	12.6	0.00	99%
MSMEG_2352		electron transfer flavoprotein, alpha subunit	etfA	1.7	11.9	0.00	100%
MSMEG_4527		ferredoxin sulfite reductase		1.8	11.4	0.00	100%
MSMEG_4261		ubiquinol-cytochrome c reductase cytochrome c subunit		1.5	12.9	0.00	99%
MSMEG_4262		ubiquinol-cytochrome c reductase iron-sulfur subunit		1.5	13.5	0.00	99%
**Energy Metabolism**							
MSMEG_5672		citrate synthase I	gltA	1.4	12.2	0.00	99%
MSMEG_5415		enolase	eno	1.8	12.2	0.00	100%
MSMEG_3084		glyceraldehyde-3-phosphate dehydrogenase, type I	gap	1.9	12.1	0.00	100%
MSMEG_1654		isocitrate dehydrogenase, NADP-dependent		1.8	11.8	0.00	98%
MSMEG_3200		L-aspartate oxidase	nadB	1.9	10	0.00	100%
MSMEG_3085		phosphoglycerate kinase	pgk	1.5	11.3	0.00	97%
MSMEG_3227		pyruvate kinase	pyk	1.2	11.7	0.00	95%
MSMEG_3199		quinolinate synthetase complex, A subunit	nadA	1.7	11	0.00	97%
MSMEG_0932		ROK family protein		2.1	11.2	0.00	100%
MSMEG_5524		succinyl-CoA synthetase, alpha subunit	sucD	1.6	10.6	0.00	99%
MSMEG_5525		succinyl-CoA synthetase, beta subunit	sucC	1.6	10.8	0.00	99%
**Protein Folding**							
MSMEG_0880		chaperonin GroL	groL	2.5	13.6	0.00	93%
MSMEG_1583		chaperonin GroL	groL	2	11.4	0.00	100%
MSMEG_1582		chaperonin GroS	groS	2.1	12.4	0.00	100%
MSMEG_0024		peptidyl-prolyl cis-trans isomerase B		3.3	13.5	0.00	100%
MSMEG_2974		peptidyl-prolyl cis-trans isomerase, cyclophilin-type		1.2	11	0.00	83%
MSMEG_3434		peptidyl-prolyl cis-trans isomerase, fkbp-type domain protein		1.1	10.7	0.00	83%
**Lipiid Metabolism**							
MSMEG_4326		acyl carrier protein	acpP	1.5	13.2	0.00	95%
MSMEG_5248		acyl-[ACP] desaturase		1.7	10.5	0.00	88%
MSMEG_2131		acyl-CoA synthase		1.5	10.1	0.00	94%
MSMEG_5273		beta-ketoadipyl CoA thiolase		1.5	11.6	0.00	99%
MSMEG_5773		fatty acid desaturase		1.4	11.4	0.00	92%
MSMEG_4351		hypothetical oxidoreductase YjgI		1.8	10.3	0.00	60%
MSMEG_0096		peroxisomal hydratase-dehydrogenase-epimerase		2.3	11	0.00	100%
**Maintenance and Growth**							
MSMEG_1843	adenosylhomocysteinase		ahcY	1.8	11.1	0.00	99%
MSMEG_1540	ATP-dependent RNA helicase			1.6	11	0.00	91%
MSMEG_6403	bifunctional udp-galactofuranosyl transferase glft			1.6	11.1	0.00	95%
MSMEG_1947	conserved hypothetical protein [(glutaredoxin)]			1.8	12.2	0.00	88%
MSMEG_4396	isochorismatase hydrolase			1.7	10	0.00	100%
MSMEG_6896	single-stranded DNA-binding protein			2.1	11.6	0.00	100%
MSMEG_4891	alkylhydroperoxide reductase			1.8	12.9	0.00	100%
MSMEG_0835	copper/zinc superoxide dismutase		sodC	1.6	11.9	0.00	97%
MSMEG_0314	glucose-6-phosphate 1-dehydrogenase		zwf	1.3	11	0.00	92%
MSMEG_4557	ABC transporter, ATP-binding protein			1.7	10.4	0.00	100%
MSMEG_4560	periplasmic binding protein			1.9	10.8	0.00	99%
MSMEG_4561	ABC Fe3+-siderophores transporter, periplasmic binding protein			1.7	9.7	0.00	94%
MSMEG_4533	sulfate-binding protein			1.7	10.5	0.00	90%
MSMEG_5788	integral membrane protein			1.6	10.4	0.00	98%
MSMEG_5789	putative thiosulfate sulfurtransferase			3.2	11.9	0.00	100%
MSMEG_5790	SseC protein			3.5	12.6	0.00	100%

**Appendix Table 3:** Select up-regulated operons and clusters in stationary phase over log phase (non-spray dried log *versus* stationary comparision).

Primary Target	Common Name	Gene	Intensity Ratio	Average Channel Intensity	p-value	Probability of Differential Expression
**Carbon Limitation**						
MSMEG_1552	ethanolamine permease	eat	1.5	12.7	0.00	72%
MSMEG_1553	ethanolamine ammonia-lyase, large subunit	eutB	2.3	11.6	0.00	95%
MSMEG_1554	ethanolamine ammonia-lyase, light chain	eutC	2.4	10.5	0.00	99%
MSMEG_1970	sigma factor		4.2	11.6	0.00	99%
MSMEG_1971	propane monooxygenase hydroxylase large subunit		3.5	11.1	0.00	99%
MSMEG_1973	propane monooxygenase hydroxylase small subunit		2.1	10.6	0.00	98%
MSMEG_1972	methane monooxygenase component C		1.4	9.9	0.00	82%
MSMEG_1974	propane monooxygenase coupling protein		2.0	10.9	0.00	99%
MSMEG_1975	amidohydrolase 2		2.3	10.8	0.00	97%
MSMEG_1976	conserved hypothetical protein		2.4	10.2	0.00	100%
MSMEG_1977	alcohol dehydrogenase		1.7	10.5	0.00	94%
MSMEG_1978	chaperonin GroL	groL	2.0	10.5	0.00	89%
MSMEG_1979	antibiotic biosynthesis monooxygenase		1.3	11.4	0.00	99%
MSMEG_4206	Molybdopterin oxidoreductase		3.1	10.8	0.00	98%
MSMEG_4207	universal stress protein family protein		3.1	11.9	0.00	99%
MSMEG_4208	integral membrane protein		4.2	12.3	0.00	100%
MSMEG_4209	integral membrane protein		3.8	11.4	0.00	99%
MSMEG_4210	secreted protein		4.9	11.6	0.00	100%
**Scavenging Pathways**						
MSMEG_1411	universal stress protein family protein		1.3	10.3	0.00	85%
MSMEG_1412	amino acid permease		1.3	11.0	0.00	91%
MSMEG_1413	ornithine--oxo-acid transaminase	rocD	2.3	10.9	0.00	94%
MSMEG_1414	Amidinotransferase		2.0	11.3	0.00	97%
MSMEG_1417	glyoxalase family protein		1.0	9.9	0.00	65%
MSMEG_1418	RNA polymerase ECF-type sigma factor		0.8	10.7	0.00	67%
MSMEG_5117	proline dehydrogenase		2.9	11.7	0.00	100%
MSMEG_5119	1-pyrroline-5-carboxylate dehydrogenase	pruA	2.8	12.0	0.00	100%
MSMEG_2748	soluble pyridine nucleotide transhydrogenase	sthA	2.4	10.8	0.00	99%
**Membrane and Cell Wall Synthesis**						
MSMEG_2522	efflux ABC transporter, permease protein		1.6	12.0	0.00	98%
MSMEG_2523	efflux ABC transporter, permease protein, putative		3.1	11.0	0.00	99%
MSMEG_2524	ABC transporter, ATP-binding protein		3.8	10.9	0.00	100%
MSMEG_2525	amino acid permease superfamily protein		4.7	12.1	0.00	99%
MSMEG_2526	copper methylamine oxidase		3.2	12.1	0.00	100%
**Oxygen Limitation**						
MSMEG_2270	hypothetical protein		1.1	10.8	0.00	80%
MSMEG_2271	hydrogenase accessory protein HypB	hypB	0.8	11.5	0.00	67%
MSMEG_2272	hydrogenase nickel insertion protein HypA	hypA	1.0	11.4	0.00	87%
MSMEG_2273	[NiFe] hydrogenase maturation protein HypF	hypF	1.6	10.6	0.00	92%
MSMEG_2274	hydrogenase assembly chaperone HypC/HupF	hypC	1.2	12.3	0.00	95%
MSMEG_2276	hydrogenase expression/formation protein HypE	hypE	0.7	14.0	0.01	34%
MSMEG_2702	hydrogenase expression/formation protein HypD	hypD	1.3	10.7	0.00	90%
MSMEG_2703	hydrogenase assembly chaperone HypC/HupF	hypC	1.5	10.3	0.00	99%
MSMEG_2705	hydrogenase expression/formation protein HypE	hypE	1.5	10.2	0.00	90%
MSMEG_2706	phosphoheptose isomerase	gmhA	2.5	10.3	0.00	100%
MSMEG_2711	[NiFe] hydrogenase maturation protein HypF	hypF	1.6	10.2	0.00	98%
MSMEG_2712	hydrogenase assembly chaperone HypC/HupF	hypC	1.7	10.8	0.00	98%
MSMEG_2713	peptidase M52, hydrogen uptake protein		2.4	10.7	0.00	100%
MSMEG_2714	hypothetical protein		2.1	10.6	0.00	100%
MSMEG_2715	conserved hypothetical protein		2.0	9.8	0.00	94%
MSMEG_2716	conserved hypothetical protein		2.1	10.2	0.00	100%
MSMEG_2718	iron-sulfur cluster-binding protein, Rieske family protein, putative		2.4	10.3	0.00	95%
MSMEG_2719	hydrogen:quinone oxidoreductase		2.7	10.8	0.00	98%
MSMEG_2720	NADH ubiquinone oxidoreductase, 20 kda subunit		2.7	11.3	0.00	99%
**Oxydative Stress**						
MSMEG_3461	catalase/peroxidase HPI	katG	2.7	11.1	0.00	97%
MSMEG_3708	catalase		2.3	10.6	0.00	82%
MSMEG_6213	Manganese containing catalase		2.0	10.8	0.00	99%
**Nitrogen Limitation**						
MSMEG_0427	nitrite reductase [NAD(P)H], large subunit	nirB	2.3	11.4	0.00	100%
MSMEG_0428	nitrite reductase [NAD(P)H] small subunit		2.9	11.0	0.00	100%
MSMEG_0429	putative ferric uptake regulator		1.1	9.6	0.00	77%
MSMEG_0431	secreted protein		1.0	10.5	0.00	67%
MSMEG_0432	uroporphyrinogen-III synthetase		1.6	9.4	0.00	88%
MSMEG_0433	nitrite extrusion protein		1.8	12.1	0.00	97%
MSMEG_0434	aminoglycoside 2'-N-acetyltransferase (AAC(2')-Id)		1.4	10.7	0.00	84%
MSMEG_0435	allophanate hydrolase subunit 2		1.8	11.8	0.00	99%
MSMEG_0436	allophanate hydrolase subunit 1		1.2	12.6	0.00	96%
**Other Stress Related**						
MSMEG_1597	Transcription factor WhiB		1.5	9.9	0.00	86%
MSMEG_1764	L-lysine-epsilon aminotransferase		2.2	11.2	0.00	99%
MSMEG_1769	UsfY protein		0.8	10.7	0.00	78%
MSMEG_1787	RsbW protein		0.8	10.2	0.00	74%
MSMEG_1791	UsfY protein		1.5	11.1	0.00	99%
MSMEG_6467	starvation-induced DNA protecting protein		1.5	12.8	0.06	2%
MSMEG_1953	transcription factor WhiB		1.0	11.6	0.00	62%

**Appendix Table 4:** Differential gene expression of sigma factors (non-spray dried log *versus* stationary comparison).

Primary Target	Common Name	Intensity Ratio	Average Channel Intensity	p-value	Probability of Differential Expression
**Stationary Phase (Positive Intensity Ratio)**					
MSMEG_1970	sigma factor	4.2	11.6	0.00	99%
MSMEG_1599	RNA polymerase sigma-70 factor	1.9	10.9	0.00	98%
MSMEG_6817	RNA polymerase sigma factor, sigma-70 family protein	1.3	9.7	0.00	90%
MSMEG_3008	putative sigma 54 type regulator	1.1	9.6	0.00	75%
MSMEG_1418	RNA polymerase ECF-type sigma factor	0.8	10.7	0.00	64%
MSMEG_0219	RNA polymerase sigma-70 factor, family protein	0.8	10.4	0.02	9%
MSMEG_1690	putative ECF sigma factor RpoE1	0.5	9.2	0.03	15%
MSMEG_1666	RNA polymerase sigma-70 factor	0.5	9.3	0.03	9%
MSMEG_1486	RNA polymerase sigma-70 factor	0.4	9.7	0.06	2%
MSMEG_5444	RNA polymerase sigma-70 factor	0.4	9.7	0.05	2%
MSMEG_1914	RNA polymerase sigma-70 factor, family protein	0.4	11.3	0.10	1%
MSMEG_5214	RNA polymerase sigma-70 factor	0.2	9.5	0.34	0%
MSMEG_5072	extracytoplasmic function alternative sigma factor	0.2	11.9	0.33	0%
MSMEG_3296	ECF-family protein sigma factor H	0.2	9.2	0.26	0%
MSMEG_1692	ECF-family protein RNA polymerase sigma factor	0.1	9.3	0.47	0%
MSMEG_4315	RNA polymerase sigma factor, sigma-70 family protein	0.1	10.4	0.66	0%
MSMEG_0574	putative ECF sigma factor RpoE1	0.0	9.3	0.57	0%
**Log Phase (Negative Intensity Ratio)**					
MSMEG_5365	RNA polymerase sigma-70 factor	-0.8	10.1	0.00	59%
MSMEG_0405	extra cytoplasmic sigma factor	-0.6	10.1	0.01	38%
MSMEG_1804	RNA polymerase sigma-F factor	-0.3	11.2	0.09	1%
MSMEG_1348	RNA polymerase ECF-subfamily protein sigma factor	-0.3	9.1	0.24	0%
MSMEG_3485	putative ECF sigma factor RpoE1	-0.3	9.6	0.21	0%
MSMEG_2758	sigma factor MysA	-0.2	12.0	0.19	0%
MSMEG_6931	RNA polymerase sigma-70 factor	-0.2	9.5	0.37	0%
MSMEG_0573	putative ECF sigma factor RpoE1	-0.2	10.8	0.44	0%
MSMEG_2752	sigma factor SigB	-0.2	12.8	0.39	0%
MSMEG_4405	putative ECF sigma factor RpoE1	-0.1	9.3	0.60	0%
MSMEG_6682	RNA polymerase sigma-70 factor, putative	-0.1	8.9	0.68	0%
MSMEG_1747	RNA polymerase sigma-70 factor	-0.1	9.1	0.55	0%
MSMEG_3275	RNA polymerase sigma factor, sigma-70 family protein	-0.1	9.0	0.61	0%

**Appendix Table 5:** Log phase differential gene expression in multiply cycled bacteria *versus* non-previously spray dried bacteria (positive expression indicates stationary phase).

Primary Target	Common Name	Gene	Intensity Ratio	Average Channel Intensity	p-value	Probability of Differential Expression
MSMEG_0267	esterase		0.6	9.1	0.047	53%
MSMEG_0451	oxidoreductase, FAD-linked		0.7	10.9	0.017	84%
MSMEG_0536	intracellular protease, PfpI family protein		0.8	10.4	0.008	93%
MSMEG_0670	FAD dependent oxidoreductase		0.5	9.4	0.043	57%
MSMEG_0671	S-(hydroxymethyl)glutathione dehydrogenase		0.7	10.1	0.014	91%
MSMEG_0672	conserved hypothetical protein		1.4	12.6	0.027	68%
MSMEG_0685	oxidoreductase, molybdopterin-binding subunit		0.7	10.0	0.020	89%
MSMEG_1076	conserved hypothetical protein		2.0	12.3	0.002	100%
MSMEG_1097	glycosyl transferase, group 2 family protein		1.5	11.6	0.002	100%
MSMEG_1131	tryptophan-rich sensory protein		1.1	10.6	0.032	70%
MSMEG_1558	conserved hypothetical protein		0.8	9.5	0.032	73%
MSMEG_1605	phosphate transport system regulatory protein PhoU	phoU	0.6	9.3	0.009	91%
MSMEG_1766	conserved hypothetical protein		0.8	9.1	0.026	78%
MSMEG_1767	conserved hypothetical protein		0.9	10.1	0.004	98%
MSMEG_1768	conserved hypothetical protein		0.9	9.2	0.012	96%
MSMEG_1769	UsfY protein		1.3	9.6	0.002	100%
MSMEG_1770	conserved hypothetical protein		1.7	10.7	0.001	100%
MSMEG_1771	methylase, putative		1.3	10.9	0.006	95%
MSMEG_1772	conserved hypothetical protein		1.5	9.9	0.005	99%
MSMEG_1773	conserved hypothetical protein		1.1	10.7	0.007	95%
MSMEG_1774	conserved hypothetical protein		1.2	11.5	0.020	76%
MSMEG_1777	UsfY protein		1.3	11.5	0.005	97%
MSMEG_1782	oxidoreductase, short chain dehydrogenase/reductase family protein		0.7	10.6	0.021	87%
MSMEG_1783	hypothetical protein		0.6	9.6	0.042	78%
MSMEG_1788	conserved hypothetical protein		1.4	10.7	0.003	98%
MSMEG_1789	conserved hypothetical protein		1.4	10.8	0.007	93%
MSMEG_1790	conserved hypothetical protein		1.2	9.5	0.005	98%
MSMEG_1792	conserved hypothetical protein		0.5	9.5	0.046	55%
MSMEG_1794	dehydrogenase		0.6	9.4	0.020	81%
MSMEG_1802	ChaB protein		0.8	9.2	0.033	89%
MSMEG_1821	acyl-CoA dehydrogenase		**-0.6**	9.4	0.017	79%
MSMEG_1886	Fatty acid desaturase		0.9	9.9	0.012	88%
MSMEG_1950	conserved hypothetical protein		1.0	10.2	0.004	98%
MSMEG_1951	conserved domain protein		1.7	10.5	0.003	99%
MSMEG_1952	ATP-dependent DNA helicase		0.6	9.7	0.029	76%
MSMEG_2112	secreted protein		1.0	10.1	0.006	96%
MSMEG_2115	conserved hypothetical protein		1.0	9.7	0.004	99%
MSMEG_2345	lycopene cyclase		0.7	8.7	0.042	75%
MSMEG_2346	phytoene synthase		1.0	9.0	0.003	99%
MSMEG_2376	conserved hypothetical protein		0.7	9.1	0.018	79%
MSMEG_2415	hemerythrin HHE cation binding region		1.1	10.3	0.003	99%
MSMEG_2593	gnat-family protein acetyltransferase		0.9	10.1	0.005	98%
MSMEG_2594	asparagine synthase (glutamine-hydrolyzing)	asnB	0.8	9.9	0.010	92%
MSMEG_2913	hydrolase		0.6	9.2	0.021	76%
MSMEG_2924	permease binding-protein component		0.7	10.2	0.021	90%
MSMEG_2925	permease membrane component		0.9	8.9	0.008	92%
MSMEG_2926	glycine betaine/carnitine/choline transport ATP-binding protein opuCA		1.0	10.1	0.006	98%
MSMEG_2958	conserved hypothetical protein		1.0	10.3	0.003	98%
MSMEG_3022	transglycosylase associated protein		1.6	10.5	0.008	94%
MSMEG_3184	malto-oligosyltrehalose trehalohydrolase	treZ	0.8	9.4	0.012	90%
MSMEG_3185	putative maltooligosyl trehalose synthase		0.8	10.2	0.006	95%
MSMEG_3186	glycogen debranching enzyme GlgX	glgX	0.6	10.8	0.041	77%
MSMEG_3254	RDD family protein, putative		1.0	10.6	0.004	98%
MSMEG_3255	DoxX subfamily protein, putative		1.3	10.1	0.002	100%
MSMEG_3418	conserved hypothetical protein		0.8	9.7	0.009	95%
MSMEG_3419	hypothetical protein		1.2	10.3	0.006	96%
MSMEG_3543	soluble secreted antigen MPT53		0.8	9.5	0.044	94%
MSMEG_4325	malonyl CoA-acyl carrier protein transacylase		**-**0.9	12.1	0.020	88%
MSMEG_4918	1,4-alpha-glucan branching enzyme	glgB	0.7	10.3	0.022	75%
MSMEG_4991	hypothetical protein		0.7	9.8	0.017	83%
MSMEG_4993	hypothetical protein		1.0	10.3	0.007	93%
MSMEG_5342	conserved hypothetical protein		0.9	9.1	0.018	89%
MSMEG_5343	conserved hypothetical protein		1.1	9.2	0.010	98%
MSMEG_5542	transcriptional regulator, HTH_3 family protein		1.0	9.4	0.008	93%
MSMEG_5543	hypothetical protein		1.8	10.4	0.002	99%
MSMEG_5616	glyoxalase/bleomycin resistance protein/dioxygenase		0.7	8.7	0.017	89%
MSMEG_5617	immunogenic protein MPT63		1.6	10.6	0.004	99%
MSMEG_5722	conserved hypothetical protein		0.6	9.5	0.038	71%
MSMEG_5936	conserved hypothetical protein		1.1	10.9	0.003	98%
MSMEG_6211	hypothetical protein		0.8	10.5	0.011	93%
MSMEG_6212	hemerythrin HHE cation binding domain subfamily protein, putative		1.0	10.9	0.025	98%
MSMEG_6213	Manganese containing catalase		1.0	9.1	0.011	94%
MSMEG_6232	catalase KatA	katA	0.7	8.8	0.030	73%
MSMEG_6305	conserved hypothetical protein		0.6	9.1	0.027	74%
MSMEG_6355	hypothetical protein		0.7	9.1	0.024	78%
MSMEG_6467	starvation-induced DNA protecting protein		1.6	10.5	0.002	99%
MSMEG_6507	glycogen debranching enzyme GlgX	glgX	0.9	10.2	0.013	97%
MSMEG_6579	conserved hypothetical protein		0.6	10.6	0.026	69%
MSMEG_6616	S-(hydroxymethyl)glutathione dehydrogenase		0.7	9.9	0.014	87%

**Appendix Table 6:** Stationary phase differential gene expression in multiply cycled bacteria *versus* non-previously spray dried bacteria (positive expression indicates stationary phase).

Primary Target	Common Name	Gene	Intensity Ratio	Average Channel Intensity	Probability of Differential Expression
MSMEG_0536	intracellular protease, PfpI family protein		0.9	11.6	60%
MSMEG_0641	binding-protein-dependent transport systems inner membrane component		**-0.6**	10.4	21%
MSMEG_0671	S-(hydroxymethyl)glutathione dehydrogenase		1.1	10.9	27%
MSMEG_0696	alanine-rich protein		0.6	9.8	21%
MSMEG_1112	aconitate hydratase, putative		1.0	10.2	25%
MSMEG_1679	AmiB		**-0.9**	10.8	30%
MSMEG_1683	cytosine/purine/uracil/thiamine/allantoin permease family protein		**-0.7**	12.0	39%
MSMEG_1767	conserved hypothetical protein		1.3	10.8	49%
MSMEG_1768	conserved hypothetical protein		0.8	9.7	37%
MSMEG_1769	UsfY protein		1.0	10.0	56%
MSMEG_1772	conserved hypothetical protein		1.1	10.3	40%
MSMEG_1790	conserved hypothetical protein		1.4	10.4	62%
MSMEG_1950	conserved hypothetical protein		1.1	10.7	33%
MSMEG_1951	conserved domain protein		2.3	11.5	34%
MSMEG_2115	conserved hypothetical protein		2.1	11.2	61%
MSMEG_2346	phytoene synthase		1.5	10.3	66%
MSMEG_2347	phytoene dehydrogenase		0.8	11.0	21%
MSMEG_2389	DNA-binding protein HU	hup	**-0.9**	11.4	22%
MSMEG_2752	sigma factor SigB		1.2	11.2	37%
MSMEG_2958	conserved hypothetical protein		1.2	11.9	28%
MSMEG_3185	putative maltooligosyl trehalose synthase		0.7	10.9	28%
MSMEG_3254	RDD family protein, putative		1.7	11.9	28%
MSMEG_3439	hypothetical protein		2.4	12.2	25%
MSMEG_4208	integral membrane protein		**-0.9**	10.8	60%
MSMEG_5152	hypothetical protein		**-0.8**	11.9	27%
MSMEG_5542	transcriptional regulator, HTH_3 family protein		1.4	10.1	55%
MSMEG_6213	Manganese containing catalase		1.0	10.3	55%
MSMEG_6242	alcohol dehydrogenase, iron-containing		**-1.3**	12.4	51%
MSMEG_6579	conserved hypothetical protein		1.1	11.5	21%
MSMEG_6759	glycerol kinase	glpK	**-1.2**	12.4	61%
MSMEG_6761	glycerol-3-phosphate dehydrogenase 2		**-1.7**	12.9	33%

**Appendix Table 7:** Differential gene expression in non-previously spray dried bacteria in dry powder state.

Primary Target	Common Name	Gene	Intensity Ratio	Average Channel Intensity	p-value	Probability of Differential Expression
MSMEG_0114	extracellular solute-binding protein, family protein 3		1.6	8.7	0.008	95%
MSMEG_0373	3-ketoacyl-CoA thiolase		1.3	12.4	0.008	89%
MSMEG_0614	methyltransferase		1.4	9.9	0.010	74%
MSMEG_1130	hypothetical protein		2.1	7.8	0.009	77%
MSMEG_1452	sulfatase-modifying factor 1		2.8	8.3	0.008	90%
MSMEG_1479	methyltransferase, putative, family protein		1.1	9.8	0.010	73%
MSMEG_1482	methyltransferase		1.4	10.9	0.010	68%
MSMEG_1530	integral membrane protein		1.8	10.2	0.008	92%
MSMEG_1843	adenosylhomocysteinase	ahcY	1.2	10.3	0.009	82%
MSMEG_1887	hypothetical protein		1.3	12.2	0.010	71%
MSMEG_1888	methyltransferase		1.6	8.4	0.008	92%
MSMEG_1911	catechol 1,2-dioxygenase	catA	1.4	10.4	0.009	83%
MSMEG_1960	hypothetical protein		2.0	8.2	0.010	73%
MSMEG_2081	putative acyl-CoA dehydrogenase		1.4	12.6	0.009	81%
MSMEG_2316	monooxygenase, NtaA/SnaA/SoxA family		1.2	10.3	0.009	82%
MSMEG_2507	IclR-family protein transcriptional regulator		2.0	8.0	0.010	74%
MSMEG_2511	siderophore utilization protein		1.1	10.7	0.009	74%
MSMEG_2748	soluble pyridine nucleotide transhydrogenase	sthA	4.1	8.5	0.008	96%
MSMEG_2799	phospho-2-dehydro-3-deoxyheptonate aldolase		1.4	9.6	0.009	85%
MSMEG_3215	ABC-type molybdenum transport system, ATPase component/photorepair protein PhrA		1.3	10.4	0.008	84%
MSMEG_3233	cytochrome D ubiquinol oxidase subunit 1		1.3	10.6	0.009	78%
MSMEG_3724	coenzyme PQQ biosynthesis protein B	pqqB	2.1	7.6	0.009	76%
MSMEG_3902	ATPase,AAA family protein		1.4	12.2	0.010	74%
MSMEG_4085	nitrilotriacetate monooxygenase component A		1.2	8.8	0.009	80%
MSMEG_4107	Phosphoglycerate mutase, putative		2.1	7.9	0.009	77%
MSMEG_4372	capreomycidine hydroxylase		2.3	7.0	0.008	84%
MSMEG_4576	SpfH domain protein		1.1	8.0	0.009	73%
MSMEG_5005	LprC protein		1.1	8.9	0.010	72%
MSMEG_5216	glyoxalase family protein		1.7	8.3	0.008	89%
MSMEG_5226	exodeoxyribonuclease VII, large subunit	xseA	1.1	9.0	0.009	76%
MSMEG_5364	amidohydrolase 2		1.2	10.7	0.010	71%
MSMEG_5486	peptidase S1 and S6, chymotrypsin/Hap		1.7	13.1	0.008	95%
MSMEG_5646	conserved hypothetical protein		2.0	11.1	0.008	92%
MSMEG_5745	gas vesicle synthesis protein		2.0	8.3	0.010	73%
MSMEG_5789	putative thiosulfate sulfurtransferase		1.2	11.2	0.009	78%
MSMEG_5861	cytochrome P450 109		2.0	8.2	0.009	76%
MSMEG_5887	intersectin-EH binding protein Ibp1		1.2	11.8	0.010	70%
MSMEG_5912	succinic semialdehyde dehydrogenase		1.4	9.6	0.008	92%
MSMEG_5982	UDP-glucose 6-dehydrogenase		2.9	7.7	0.008	91%
MSMEG_6254	hypothetical protein		1.5	10.1	0.009	88%
MSMEG_6332	amino acid ABC transporter, permease protein		2.0	7.6	0.010	73%
MSMEG_6454	conserved hypothetical protein		1.6	11.9	0.008	92%
MSMEG_6512	acyl-CoA dehydrogenase domain protein		1.5	11.4	0.008	86%

**Appendix Table 8:** Differential gene expression in multiply cycled bacteria in dry powder state.

Primary Target	Common Name	Gene	Intensity Ratio	Average Channel Intensity	p-value	Probability of Differential Expression
**Lipid Synthesis**						
MSMEG_2337	isopentenyl-diphosphate delta-isomerase, type 2	fni	1.23	12.2	0.010	65%
MSMEG_4326	acyl carrier protein	acpP	1.58	10.5	0.008	89%
MSMEG_4327	3-oxoacyl-[acyl-carrier-protein] synthase 1		1.37	10.6	0.010	67%
MSMEG_4328	3-oxoacyl-[acyl-carrier-protein] synthase 2		1.79	10.5	0.009	87%
MSMEG_4329	propionyl-CoA carboxylase beta chain		1.78	10.6	0.008	91%
MSMEG_5242	acyltransferase, ws/dgat/mgat subfamily protein		1.68	10.4	0.009	83%
**Transposases**						
MSMEG_1862	transposase		1.04	10.2	0.012	49%
MSMEG_2824	IS1549, transposase		1.24	10.5	0.019	27%
MSMEG_4522	ISMsm2, transposase		1.37	11.1	0.008	91%
MSMEG_4072	ISMsm5, transposase		1.40	10.1	0.009	84%
MSMEG_2805	ISMsm5, transposase		1.43	11.4	0.008	88%
MSMEG_2830	ISMsm4, transposase		1.44	10.8	0.009	84%
MSMEG_4791	IS1096, tnpR protein		1.61	11.3	0.010	70%
MSMEG_3341	Transposase IS116/IS110/IS902 family protein		1.62	10.3	0.008	95%
MSMEG_3984	Transposase IS116/IS110/IS902 family protein		1.99	11.0	0.010	82%
MSMEG_1731	IS6120, transposase		2.05	10.4	0.008	94%
MSMEG_4926	IS1096, tnpA protein		2.24	12.3	0.008	98%
**IS1096 Related**						
MSMEG_6696	hypothetical protein		1.91	11.2	0.008	98%
MSMEG_0803	hypothetical protein		1.90	12.8	0.008	98%
MSMEG_0396	hypothetical protein		1.87	12.1	0.008	96%
MSMEG_1259	hypothetical protein		1.16	10.8	0.011	67%
**Other**						
MSMEG_0051	transcription factor WhiB family protein		1.70	10.3	0.008	93%
MSMEG_1599	RNA polymerase sigma-70 factor		1.12	10.9	0.009	73%
MSMEG_2294	DNA polymerase IV	dinB	1.72	10.3	0.009	90%

**Appendix Table 9:** Probability of expression of the carotenoid biosynthesis operon across the different experiments.

		Naïve Stationary *vs.* Naïve Log	Cycled Log *vs.* Naive Log	Cycled Stationary *vs.* Naive Stationary	Cycled Powder *vs.* Naive Powder	
Primary Target	Common Name of Primary Target	Probability of Differential Expression			σ above median	
MSMEG_2343	methylesterase	1%	8%	19%	0%	2.5
MSMEG_2344	dehydrogenase	1%	35%	14%	1%	2.7
MSMEG_2345	lycopene cyclase	0%	75%	17%	10%	2.3
MSMEG_2346	phytoene synthase	5%	99%	66%	0%	2.7
MSMEG_2347	phytoene dehydrogenase	0%	55%	21%	1%	1.9

**Appendix Table 10:** Results of sigF consensus promoter analysis.

				Naïve Stationary *vs.* Naïve Log			Cycled Log *vs.* Naive Log			Cycled Stationary *vs.* Naive Stationary			Cycled Powder *vs.* Naive Powder		
Primary Target	Common Name of Primary Target	Promoter Sequence	BP	FC	σ	P	FC	σ	P	FC	σ	P	FC	σ	P
		−35 −10													
MSMEG_1804	RNA polymerase sigma-F factor	GCCGTGGTTATCTCCACGTCCACGGTGTGTAT	−156	−0.3	1.2	1%	0.1	0.5	0%	0.3	0.7	3%	0.1	2.4	0%
MSMEG_0451	oxidoreductase, FAD−linked	TGACCGGTTTGGTGAGCGCGTAAAGCGGTTAT	−15	0.6	1.7	1%	0.7	1.8	84%	0.6	2.2	3%	0.1	3.5	0%
MSMEG_0670	FAD dependent oxidoreductase	CCTGAGGGTTCGACCGGCCGCATTGGGGGTAT	−16	0.7	−0.2	21%	0.5	0.1	57%	0.3	0.3	4%	0.9	2.0	27%
MSMEG_0671	S−(hydroxymethyl)glutathione dehydrogenase	ACCGGCCGTTTCAGCGGCTGCGCGTGGGGTAC	−54	0.2	0.7	0%	0.7	1.0	91%	1.1	1.7	27%	0.2	3.0	0%
MSMEG_0672	conserved hypothetical protein	CGACCGGGTTTGGCCGTCCCCACCGCGGGTAC	−57	1.1	3.5	1%	1.4	3.7	68%	1.6	4.3	8%	0.3	5.5	1%
MSMEG_0686	oxidoreductase	GACCGGCGTTTGGGCAGTGCCCGCCGGGGTAC	−15	0.2	2.1	0%	0.4	1.2	10%	0.3	2.3	1%	0.7	2.9	26%
MSMEG_1076	conserved hypothetical protein	GCGGAGGTTTCGTCCGTACCGACGAAGGGTAT	−57	0.9	2.4	1%	2.0	3.3	100%	0.7	2.4	4%	0.9	4.9	9%
MSMEG_1605	phosphate transport system regulatory protein PhoU	AACCTCGATTGAAGGGCCCCTCGGATGGGTAC	−56	1.2	0.6	83%	0.6	0.1	91%	0.4	0.0	4%	0.2	2.3	0%
MSMEG_1742	oxidoreductase	CCGCGACGTTTCGGATCGTCGTGTTCGGGTAC	−96	1.3	2.6	34%	0.4	0.5	4%	0.2	0.7	1%	0.1	5.1	0%
MSMEG_1758	hypothetical protein	AGCCCGGTTTCACCACGGTGTTCGCCGGGTAG	−15	0.9	1.0	85%	0.6	0.5	35%	0.5	1.6	1%	1.1	2.1	66%
MSMEG_1770	conserved hypothetical protein	GATCACGTTTCGGAACCCGGAATACCGGGCAT	−71	2.1	1.5	97%	1.7	1.6	100%	2.1	2.7	6%	0.3	5.5	1%
MSMEG_1771	methylase, putative	TCGGAAGGTTTGCGCGCCCGCGAGATGGGTAC	−36	1.1	2.5	1%	1.3	1.8	95%	1.6	2.8	11%	0.6	4.8	11%
MSMEG_1773	conserved hypothetical protein	GTTTGAGGTTTACCGCAGGCACAAATGGGAAT	−18	0.9	1.9	0%	1.1	1.6	95%	1.1	1.7	3%	0.1	4.2	0%
MSMEG_1774	conserved hypothetical protein	GACGGCGTTTCGCCGGGAGGCGGCCCGGGTAG	−71	0.3	2.3	0%	1.2	2.4	76%	1.3	2.3	5%	0.0	4.9	0%
MSMEG_1777	UsfY protein	GCCCGGGTTTCACACCGACCGTCCCCGGGTAG	−76	0.9	2.7	0%	1.3	2.5	97%	1.2	3.4	10%	0.4	5.7	1%
MSMEG_1794	dehydrogenase	TGCTCGTGTTCGGGGTCATATCTGGCGGGTAC	−22	1.1	0.3	47%	0.6	0.1	81%	0.4	0.4	1%	0.0	2.4	0%
MSMEG_1802	ChaB protein	TCGAGGGTTTCCCGAATGCCGACCTTGGGCAT	−70	1.1	1.4	7%	0.8	0.0	89%	0.4	0.8	2%	-0.3	1.8	0%
MSMEG_2112	secreted protein	AATTGACGTTTCTGTAGGACGCCAGCGGGTAT	−31	1.2	1.6	18%	1.0	0.9	96%	1.7	3.2	15%	0.3	4.6	1%
MSMEG_2347	phytoene dehydrogenase	CCGGACGTTTGTAGCCCGCCGCCTGCGGGTAT	−104	0.2	1.7	0%	0.6	0.9	55%	0.8	1.8	21%	0.4	1.9	1%
MSMEG_2415	hemerythrin HHE cation binding region	CTCAACGGTTGAACCCGGCCGGTAGGGGGTAG	−68	1.6	1.5	17%	1.1	1.1	99%	1.1	1.2	10%	0.6	4.5	10%
MSMEG_2958	conserved hypothetical protein	CACGACGGTTCGCCAGGTCGCCGCGCGGGTAT	−31	1.4	1.3	96%	1.0	1.1	98%	1.2	3.1	28%	0.1	5.1	0%
MSMEG_3022	transglycosylase associated protein	GCCGCCGTTTACGCCGCCGACAGCCGGGGTAT	−37	1.7	2.8	34%	1.6	1.4	94%	2.4	3.3	7%	-0.4	4.5	2%
MSMEG_3289	gp61 protein	CCTTGACGTTTGAACGTGCAGCGGGAGGGTAC	−36	1.1	1.7	96%	0.5	0.7	8%	0.4	1.1	4%	1.2	3.3	36%
MSMEG_3443	hypothetical protein	GAACGCGTTTGTCCGAGCGTCGCTGGGGATAT	−50	1.6	0.9	100%	0.5	−0.6	34%	0.6	0.4	2%	-0.1	2.4	0%
MSMEG_3543	soluble secreted antigen MPT53	CGCACGGTTCCTACCGTCGTGCCACAGGGTGT	−52	1.2	0.8	97%	0.8	0.2	94%	0.4	0.9	5%	0.0	2.8	0%
MSMEG_4072	ISMsm5, transposase	TAATTAGTTTACAGTGTGGGATGATGGTGTAT	−19	−0.8	2.0	45%	-0.1	2.8	0%	0.4	4.2	1%	1.4	1.4	84%
MSMEG_4791	IS1096, tnpR protein	TCAGCTGCTTTCGCGCTGTGATCGAGGGGTCT	−59	-0.4	4.7	2%	-0.2	0.8	0%	0.4	5.2	1%	1.6	2.3	95%
MSMEG_4918	1,4−alpha−glucan branching enzyme	ACTTTGTGGTTGGACATGGAGGCACTGGGTAT	−179	0.0	1.1	0%	0.7	1.2	75%	0.4	1.1	1%	-0.8	2.9	32%
MSMEG_5189	oxidoreductase	GGCGGCGGTTGCCGCGATCGATGCGGGGGTAT	−32	0.6	1.0	19%	0.5	0.5	48%	0.5	0.6	10%	1.1	3.0	69%
MSMEG_5343	conserved hypothetical protein	CCTGAGGTTTCACGCGTTCGCCGGATGGCTAT	−41	1.3	0.6	89%	1.1	−0.1	98%	0.4	0.1	1%	-0.6	3.2	9%
MSMEG_5543	hypothetical protein	TGTGCGTTTCGACATGCGTGAAGGCTGGGTAG	−84	0.5	2.2	0%	1.8	1.3	99%	2.4	3.0	11%	-0.5	4.0	3%
MSMEG_5617	immunogenic protein MPT63	TACCGATGTTTTCCTCCTGACGAGGCGGGTAT	−77	-0.2	0.2	0%	1.6	1.5	99%	0.5	−0.1	3%	0.5	1.8	3%
MSMEG_5872	DNA-binding response regulator PhoP	CGTGGGTTTCGGGCGGCTTCCTGCCGGGGTAT	−78	1.1	2.0	24%	0.1	1.7	0%	-0.2	1.9	0%	-0.4	5.3	1%
MSMEG_6212	hemerythrin HHE cation binding domain subfamily protein, putative	ACGCGCTGTTTGGCAACGGGTCTGACGGGTAT	−58	1.6	1.7	70%	1.0	1.8	98%	1.7	3.6	13%	0.2	5.2	0%
MSMEG_6213	Manganese containing catalase	GACCGCTGTTTGGGGTTCTCGGCGCTGGGTAT	−47	2.0	0.7	99%	1.0	−0.1	94%	1.0	0.9	55%	-0.1	4.5	0%
MSMEG_6467	starvation−induced DNA protecting protein	CGCTGTGATTAGTGCCCGGCACTGCCGGGTAC	−43	1.5	2.7	2%	1.6	1.3	99%	1.7	2.4	11%	0.3	4.7	1%
MSMEG_6616	S-(hydroxymethyl)glutathione dehydrogenase	GGCCAAGGTTTGGGCCAGCTCCGGTGGGGTAG	−37	1.2	0.9	4%	0.7	0.7	87%	1.1	2.0	6%	1.0	2.8	56%
